# Heterotopic Ossification in Hip Arthroscopy

**DOI:** 10.1055/s-0042-1758160

**Published:** 2023-02-06

**Authors:** Roberto Seijas, David Barastegui, Carlos López de Celis, Ferran Montaña, Xavier Cuscó, Ramón Cugat

**Affiliations:** 1 Instituto Cugat, Barcelona, Spain; 2 Basic Sciences Department, Faculty of Medicine and Health Sciences, Universitat International de Catalunya, Barcelona, Spain; 3 Foundation Garcia Cugat, Barcelona, Spain; 4 Mutualidad Catalana de Futbolistas, Federación Española de Fútbol, Barcelona, Spain

**Keywords:** hip, arthroscopy, heterotopic ossification, complications, surgery, NSAIDs

## Abstract

**Introduction** Hip arthroscopy is a rising surgical technique due to the increase in hip diseases, especially femoroacetabular impingement. One of the several complications related to such procedures is heterotopic ossifications (HO). The aim of this study is to describe the prevalence of HO after hip arthroscopy in a series of patients with femoroacetabular impingement and to compare its preoperative and intraoperative variables with a matched control group of patients without HO.

**Methods** All patients who underwent hip arthroscopy for femoroacetabular impingement between 2010 and 2017 with a minimum follow-up of 2 years were included in this analysis. Radiographic examinations were recorded to select cases with HO. A case-control analysis was performed comparing preoperative and intraoperative variables between cases with HO and a matched control group without HO.

**Results** A total of 700 cases were included in the analysis. HO was found in 15 (2.14%) of subjects. Cases with HO showed more severe cartilage injuries, less cam morphology ratio, and a higher proportion of partial labrectomies than the control group. No significant differences were observed in preoperative hip pain or function between groups.

**Conclusions** The prevalence of HO after hip arthroscopy in subjects with femoroacetabular impingement was 2.14%. Cases with HO had more severe cartilage injuries, lower ratio of cam morphology, and higher proportion of partial labrectomies than the control cases without HO.

**Level of Evidence** Level III.

Heterotopic ossification (HO) is defined as the abnormal bone growth in the nonskeletal soft tissue such as muscle or tendons. 1 Published studies on its incidence in hip arthroscopies highlight important differences in the results. HO has been observed in 0 to 44% of hip arthroscopies and 2 to 90% of total hip replacements. 1 2 3 4 5 6 7 8 9 10 While most of the patients with HO remain asymptomatic, patients with large ossifications or concomitant femoroacetabular impingement can present stiffness and discomfort, for which surgical treatment is indicated. 1 11 12 13

HO etiology can be genetic such as the bone morphologic protein (BMP) dysregulation or nongenetic due to direct trauma or neurological injuries. 14 15 The release of BMP in soft tissues can increase the tissue inflammatory response, which leads to modification in the biological cellular environment. Such tissular response triggers a misdifferentiation of the multipotent stem cells to osteoblasts and its consequent synthesis of ectopic tissue. 1 15 16

Several risk factors for HO have been described including male gender, previous presence of HO, advanced age, anterolateral surgical approach, presence of cerebral or spine trauma, and hip arthritis. 1 12 13 Thus, the aim of this study is to describe the prevalence of HOs after hip arthroscopy in a series of patients with femoroacetabular impingement and to compare its preoperative and intraoperative variables with a matched control group of patients without HOs.

## Methods

### Study Design

This study was a retrospective analysis of a prospective case series with a case-control analysis of the preoperative and intraoperative variables of patients with and without HO after hip arthroscopy for femoroacetabular impingement.

### Participants

From 2010 to 2017, all patients that required hip arthroscopy for femoroacetabular impingement were systematically included in a prospective database. Those who had a follow-up period more than 2 years were approached for eligibility.

All subjects conducted a baseline assessment consisting of the visual analog scale (VAS) for hip pain, 17 the modified Harris hip score (mHHS), 18 the hip outcome score (HOS), 19 and the international hip outcome test 33 (iHOT-33). 20 The assessments and a radiological control were repeated at 3, 6, 12, and 24 months after the surgery. Additionally, several intraoperative outcomes were recorded in the database including femoroacetabular impingement (FAI) morphology, which could be cam, pincer or mixed, presence of a labrum tear, treatment of labrum tear, and the acetabular labral articular disruption cartilage injury grade.

Radiographic exams were reviewed to obtain the number of patients with HO by the Brooker classification. 21 Cases were defined as Brooker grade ≥ I. For the case-control analysis, a control group was generated by randomly selecting 50 subjects with a Brooker grade = 0 matched by age and BMI to the case group. Preoperative and intraoperative variables from both groups were compared. All patients gave informed consent and allowed the authors to include their data in the prospective database.

### Surgical Technique

Facilities, anesthetic and surgical team, postsurgical follow-up, and pharmacological guidelines were the same for all cases. The surgical procedure was performed using a classic approach. Three standard lateral, distal anterolateral, and anterior portals were used. Interportal capsulotomy was not performed routinely, as well as the vertical capsular approach, which was only performed in those cases in which a femoral osteoplasty was required. In all surgeries, the orbicular ligament was preserved. Once the traction was withdrawn and the hip was flexed at around 45 degrees, a femoral correction with rotations to control the anterior and posterior milling was performed. All surgeries ended without closing the articular capsule. Finally, subjects were instructed to follow a systematic prophylactic therapy to avoid deep venous thrombosis consisting of 10 days of low-molecular heparin and 50 mg of diclofenac every 8 hours for 14 days.

### Statistical Analysis

Descriptive statistics were performed for all measurements. Normal distribution of the sample was analyzed using the Shapiro–Wilk test. In cases of normal distribution, chi-square tests and unpaired *t* -tests were used for qualitative and quantitative variables, respectively. In cases of nonnormal distribution, Fisher's exact test and Mann–Whitney U tests were used instead. The significance level was set at 0.05. All analyses were performed using SPSS Statistics (IBM Corp. Released 2011. IBM SPSS Statistics for Windows, Version 20.0. Armonk, NY: IBM Corp.).

## Results

**Fig. 1:**
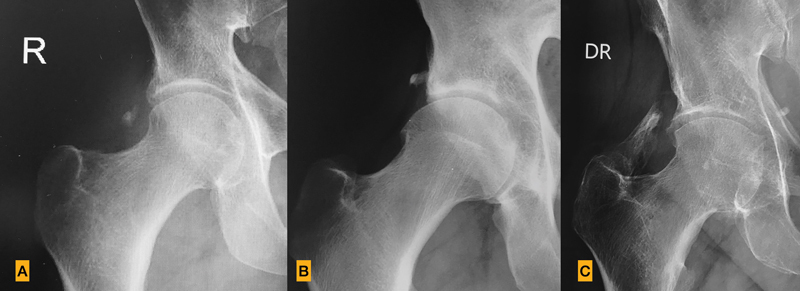
( **A** ) Brooker grade 1 of heterotopic ossifications. ( **B** ) Brooker grade 2 of heterotopic ossifications. ( **C** ) Brooker grade 3 of heterotopic ossifications.

Between 2010 and 2017, 700 patients with hip arthroscopy for FAI were recorded in the database. From those, 15 (13 males, 2 females; 2.14%) presented radiological findings of HO. According to the Brooker classification, 10 patients had an HO grade I, one patient grade II, and four patients grade III ( Fig. 1 ). None of the cases were associated with alterations in mobility, local pain, or required second surgery due to the ossification. The HOs were observed between 3- and 6-months after the surgery, with no progression in ossification noticed at 24-months.

A control group was randomly selected by choosing 50 subjects with a Brooker grade = 0 in their postoperative radiographic examinations matched to the case group by age and BMI. The characteristics of both groups are shown in Table 1 .

**Table 1:** Patient characteristics

	Heterotopic ossification (cases group) ( *n* = 15)	Control group ( *n* = 50)	*p* -Value
Sex			
Male	13 (86.7%)	34 (68.0%)	0.156
Female	2 (13.3%)	16 (32.0%)	
Age	42.73 ± 13.81	39.40 ± 13.28	0.401
Side			
Left	5 (33.3%)	19 (38%)	0.743
Right	10 (66.7%)	31 (62%)	
BMI	24.39 ± 2.61	23.89 ± 3.23	0.371

Abbreviation: BMI, body mass index.

No differences were observed in sex, age, BMI, or involved leg between groups ( *p* > 0.05).

When comparing preoperative and intraoperative variables, the HO cases showed significant differences in the distribution of FAI morphology. The HO cases showed less cam morphology proportion than the control group ( *p* = 0.022). Additionally, cases with HO showed higher rates of partial labrectomies and more severe cartilage injuries than the control group ( Table 2 ). On the contrary, between-group differences were neither observed in preoperative pain or functional outcomes (VAS, HOS adl, HOS ss, mHHS, and iHOT-33) nor in the nature of labral injuries.

**Table 2:** Between-group analysis of preoperative and intraoperative variables

	Heterotopic ossification (cases group) ( *n* = 15)	Control group ( *n* = 50)	*p* -Value
FAI morphology			
Cam	6 (40%)	38 (76%)	**0.022** a
Mixed	7 (46.7%)	10 (20%)	
Pincer	2 (13.3%)	2 (4%)	
Labrum injury			
Labrum tear	7 (46.7%)	24 (48%)	0.928
Chrondrolabral rupture	8 (53.3%)	26 (52%)	
Labrum treatment			
Suture	8 (53.3%)	43 (86%)	**0.007** a
Partial labrectomy	7 (46.7%)	7 (14%)	
ALAD			
0	0	2 (4%)	**0.031** a
1	2 (13.3%)	16 (32%)	
2	1 (6.7%)	9 (18%)	
3	3 (20%)	15 (30%)	
4	4 (60%)	8 (16%)	
VAS	55.80 ± 18.27	58.64 ± 21.56	0.646
HOS adl	76.59 ± 18.13	69.88 ± 19.74	0.245
HOS ss	54.00 ± 27.79	44.11 ± 25.81	0.206
mHHS	67.17 ± 16.19	71.65 ± 19.36	0.196
iHOT-33	38.17 ± 17.55	48.26 ± 23.58	0.141

Abbreviation: ALAD, acetabular labral articular disruption; FAI, femoroacetabular impingement; HOS, hip outcome score; HOS ADL, hip outcome scores in activities of daily living; HOS SS, hip outcome scores in sport scale; iHOT-33, international hip outcome test 33; mHHS, modified Harris hip score; VAS, visual analog scale.

Note: Bold means significant differences.

a Significance level < 0.05.

## Discussion

This study showed an HO prevalence of 2.14% after hip arthroscopy in a series of patients with femoroacetabular impingement with 2-year follow-up. It also showed that those cases with HO had lower cam morphologies, higher rates of partial labrectomies, and more severe cartilage injuries. The same preoperative levels of hip pain and function were observed in the cases with and without HO.

HO has been a usual complication related to hip surgeries for years. 8 9 10 The grade of ossification is often described by Brooker classification which ranges from grade I, the appearance of woven bone in soft tissues, to a grade IV, complete articular ankylosis.

Previous studies have reported highly variable rates of HO after hip arthroscopy, some of them reaching up to an HO prevalence of 90%. 8 9 22 23 Recently, lower prevalence rates have been reported according to miscellaneous articles. 24 25 Even though the appearance of HO is highly variable, 2 the postoperative use of specific antiinflammatory medication such as celecoxib has been proved to reduce the number of HO cases, 26 with some case series describing an HO prevalence lower than 2 and 10%. 1 2 9 25 27 The rate of HO presented in this study is low compared with previous analysis. This fact could be explained by the surgical technique performed, with a special focus on the preservation of the access portals, with no interportal capsulotomies, with the complete preservation of the orbicular ligament, and without capsulotomy or vertical minimal capsulotomy. Another possible explanation for the lower ratios of HO reported in this study could be the systematic prescription of nonsteroidal anti-inflammatory drugs (NSAIDs) after the surgery. NSAIDs lead to an alteration in the osteoprogenitor cell, modifying the cellular environment and interfering with the tissue signals. 15 As results of such effect, NSAIDs have been included in several postoperative protocols with positive reported effects on HO formation. 11 25 26 27 28 29 30 31 32 Several studies have demonstrated that the use of NSAIDs diminishes the rate of HO after hip arthroscopy. 3 4 33 Bedi et al described an important HO rate decrease from 8.1 to 1.8% with the systematic use of NSAIDs, 11 similar to the results showed by Randelli et al, with a notable decrease from 33 to 0% after the prophylactic use of NSAIDs for 3 weeks after the arthroscopic surgery. 25 However, there is a concern regarding the use of NSAIDs due to their intrinsic side effects, especially in older patients, that can cause gastrointestinal problems, bleedings or renal alterations, and blood pressure modifications. 27 In those cases, alternative preventive therapies such as low doses of radiotherapy may be useful, as they have been proved as a valid method for reducing HO rates, although at higher economic costs. 12 26

Risk factors for HOs have been widely described after total hip arthroplasties, but less attention has been given to the risk factors of HO after hip arthroscopy. 2 5 6 7 Randelli et al observed a relationship between HO and capsular incision, the excess of bone resection, the location of labral approaches, and male gender. 25 Bedi et al described male gender and osteochondroplasty performance as risk factors for HO. 11 On the contrary, Rath et al and Amar et al reported contradictory results in capsular closing as a risk factor for HO appearance. 29 34 In the current study, those HO cases reported more severe cartilage injuries and less cam morphology proportion than the control group with no HO. Additionally, HO cases presented higher rates of partial labrectomies compared with the control group in which more labral sutures were performed. Our results suggest that those patients with more severe injury grades may require associated surgical procedures that could damage the surrounding soft tissues and promote ossifications. It is known that aggressive handling during hip arthroplasties produces higher injury rates. 2 9 Further studies should investigate the relation between the number of surgical actions required, injury severity, and the appearance of HOs in larger samples with more HO cases.

This study has some limitations. First, the relation between NSAIDs use and HO rate was not studied as in the current case series all subjects underwent a postoperative prophylactic treatment that included NSAIDs for 14 days. The same limitation happened regarding the surgical technique, all subjects were operated by the same medical and anesthetic team which, although increasing the between-group homogeneity, does not allowed us to study the relation between surgical actions and HO rates.

## Conclusions

HOs were found in 2.14% of the subjects after hip arthroscopy for femoroacetabular impingement and 14 days of NSAIDs prophylactic protocol. HO cases had more severe cartilage injuries, lower proportion of cam morphologies, and higher rates of partial labrectomies compared with controls without HO.
